# Physical literacy: unlocking its inclusive potential in schools and beyond

**DOI:** 10.3389/fspor.2026.1866837

**Published:** 2026-09-16

**Authors:** Myriam Bérubé, Margaux Hebinck, Marie Laberge, Claudia Verret, Annie Pouliot-Laforte

**Affiliations:** 1 Département d'organisation et ressources humaines, École des Sciences de la gestion, Université du Québec à Montréal, Montreal, QC, Canada; 2 Département des sciences de l’activité physique, Faculté des sciences, Université du Québec à Montréal, Montreal, QC, Canada; 3 Technopôle en réadaptation pédiatrique, Centre de recherche Azrieli du CHU Sainte-Justine, Montreal, QC, Canada; 4 École de réadaptation, Faculté de médecine, Université de Montréal, Montreal, QC, Canada; 5 Groupe de recherche en activité physique adaptée (GRAPA), Faculté des Sciences, Université du Québec à Montréal, Montreal, QC, Canada

**Keywords:** adapted physical activity, ecosystemic approach, inclusivity, physical activity, physical literacy

## Abstract

Physical literacy, originally developed within physical education settings to foster lasting engagement in physical activity, is increasingly recognized in Canada as a multidimensional framework encompassing physical, cognitive, and affective dimensions that support health and participation. Despite its inclusive intent, its implementation continues to face challenges related to equity, diversity, and social justice. This paper offers a concise synthesis of the existing literature and summarizes key insights from a brainstorming activity, grounded in values of inclusion. It highlights four major challenges regarding the inclusivity of physical literacy: 1) Redefining and Validating the Concept, 2) Mobilization within Educational Settings, 3) Bridging Knowledge Mobilization Gaps, and 4) Acknowledging Cultural and Systemic Impacts; and identifies levers for action spanning multiple societal levels. We aim to emphasize the need to understand the various factors influencing inclusion within this concept and to engage diverse knowledge users in co-developing situated solutions. An ecosystemic approach that bridges health, education, and community sectors is recommended to ensure the relevance and sustainability of initiatives.

## 1 Introduction

In recent years, the concept of physical literacy, originally developed within physical education settings to promote lasting engagement in physical activity (1), has become increasingly recognized across a range of contexts, including recreational settings, and is progressively being integrated into health, rehabilitation, and governmental systems (2). Re-propelled by Margaret Whitehead (2001), physical literacy aims to reframe physical activity and related educational approaches by fostering sustained participation, enhancing motivation and confidence, and strengthening overall enjoyment (3, 4). At its core, the concept pursues holistic “human flourishing” (4). Physical literacy, as conceptualized in Whitehead's model and further endorsed by the International Physical Literacy Conference and Sport for Life, is understood as a lifelong journey encompassing physical, cognitive, and affective dimensions. Together, these dimensions contribute to a behavioral dimension reflected in an active lifestyle and human flourishing. It involves the development of fundamental movement skills across diverse environments, the acquisition of knowledge about the benefits and safe practice of physical activity, and engagement in positive experiences that foster intrinsic motivation and confidence (3, 4) (see Figure 1).

**Figure 1:**
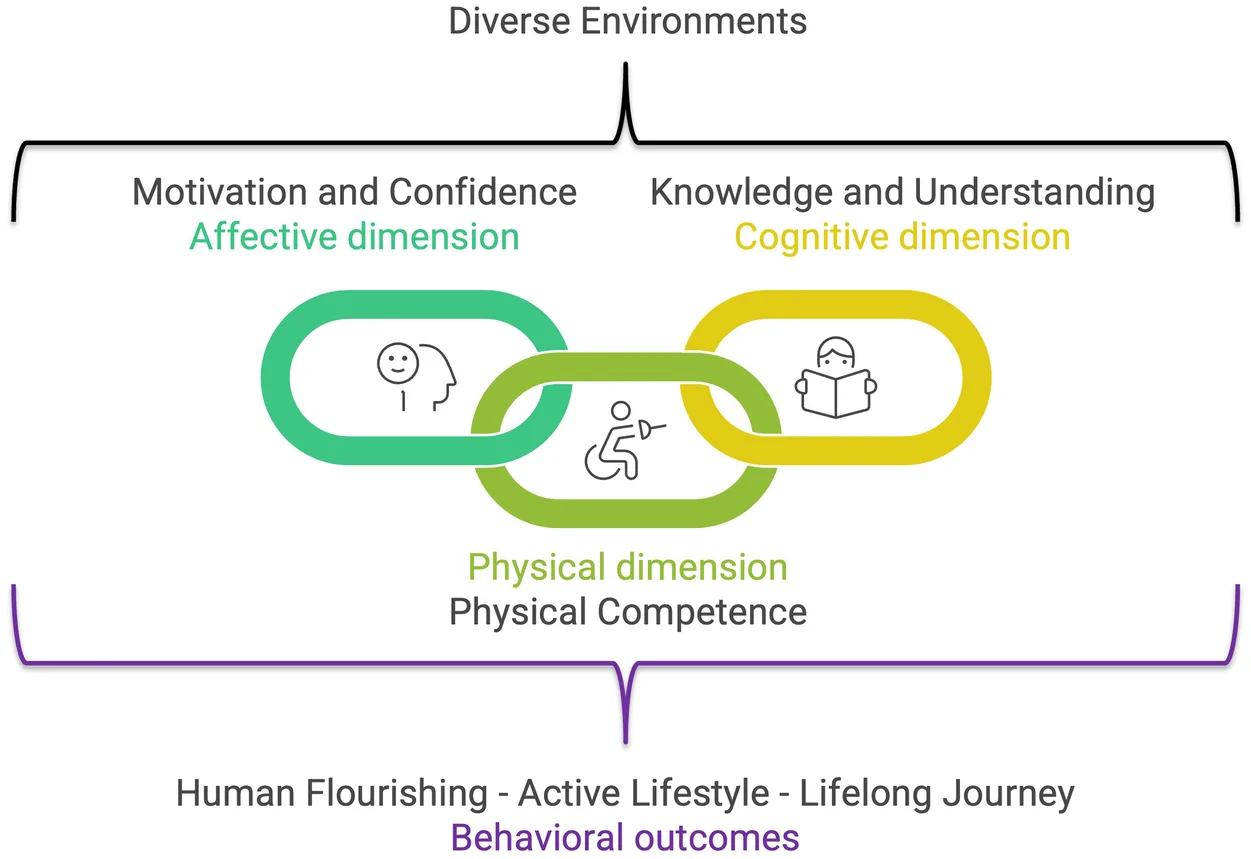
Main dimensions of the definition of physical literacy. The figure highlights the three core dimensions of physical literacy, which can be mobilized across a diversity of environments: motivation and confidence (affective dimension), knowledge and understanding (cognitive dimension), and physical competence (physical dimension). Together, they contribute to behavioral outcomes reflected in an active lifestyle, human flourishing, and engagement in a lifelong physical activity journey.

When first enunciated, the concept was presented as having both the intention and potential to foster inclusivity (4, 5). Notably, the components of physical literacy are unique to each person; they are not assessed in comparison to others but are centered on the person's own development and experience (6). This perspective aligns closely with the principles of inclusion and social justice, as it recognizes and values the diversity of abilities, backgrounds, and trajectories (4). Importantly, these trajectories are also shaped by social norms related to sex and gender, which influence access to opportunities, expectations, and experiences in physical activity contexts. For example, girls often report lower perceived competence and enjoyment in physical education compared to boys, which negatively affects their motivation and participation over time (7). Motivation patterns also differ, with girls tending to emphasize mastery and skill development, whereas boys are more likely to be driven by competition and peer comparison (8). As a result, certain activities, environments, and performance standards remain implicitly gendered.

As a society committed to equity and human rights, we must recognize the right of all individuals to explore their movement potential, regardless of age, ability, or background (9, 10). The World Health Organization identifies physical activity as a central determinant of health and well-being (11), while the United Nations Convention on the Rights of the Child explicitly affirms every child's right to engage in play and recreational activities (12). This principle is reiterated in the Canadian Sport Policy 2025–2035, which emphasizes that everyone should have the opportunity to participate in physical activity within inclusive and welcoming environments (13). Similarly, organizations such as Physical and Health Education Canada have positioned physical literacy as a central theme through practical tools and initiatives related to injury prevention, inclusion, and implementation guidelines (14). In this context, physical literacy provides a relevant framework to operationalize these inclusive principles, as highlighted by Cairney et al. (2019), who conceptualize it as a multidimensional construct supporting meaningful engagement in physical activity (15). Given its substantial promise for enhancing inclusion if appropriately implemented, physical literacy merits focused attention and action at all levels of society (9).

Currently in Canada, the implementation of physical literacy remains fraught with notable challenges, particularly in areas related to equity, diversity, and inclusion (16). While initiatives such as Sport for Life's Inclusive Physical Literacy training demonstrate a growing awareness and commitment to addressing these issues, recent literature reviews reveal the extent of the work that remains (16). Critics and researchers alike highlight persistent barriers that prevent the full realization of its inclusive potential (9, 16). Through a participatory process building on the engagement of multiple knowledge users, Robert et al. (2023) identified and prioritized challenges currently limiting the implementation of physical literacy, particularly for individuals with physical disabilities (17). They identified five gaps: (A) insufficient training of professionals in physical literacy principles; (B) the lack of a shared lifespan definition; (C) the absence of community-oriented measurement tools; (D) limited availability of normative data on fundamental movement skills; and (E) the persistent disconnect between rehabilitation services and community-based programs. Indeed, the concept is often difficult to translate into practice, given the current educational norms and readily accessible resources in communities (6, 9, 18). Although changing those structures is not easily done, we must critically examine and confront the gaps between current standards and the aspirational concept of physical literacy.

This perspective paper aims to issue a call to action to broaden physical literacy. It offers a concise synthesis of the existing literature and summarizes key insights from a brainstorming activity. The paper highlights four major challenges related to the inclusivity of physical literacy and identifies levers for action spanning multiple societal levels. Organizations could draw on these levers for action to develop tailored action plans grounded in their contexts.

## 2 Authors' perspective

The research team was composed of three researchers and two early-career scholars, including professionals trained in physiotherapy and occupational therapy with expertise in sex- and gender-based analysis, as well as specialists in kinesiology and physical education with expertise in adapted physical activity. All members share a conceptualization of disability as the result of interactions between individuals and their environments, encompassing social and institutional contexts that can support or hinder participation (19). These environments are shaped by prevailing social norms and expectations, which may reflect ableist assumptions (20). Ableism can be understood as a set of beliefs, processes, and practices that promote specific standards of body, functioning, and performance as normative and desirable (21). Such norms influence how physical activity is structured (e.g., program design, definitions of competence, and success criteria) and may contribute, often unintentionally, to forms of exclusion. The team's stance is grounded in values of inclusion in physical education (9, 16), informed by critiques of ableist norms (22) and sex- and gender-based analysis (23).

The perspective developed in this paper is grounded in both existing literature and the authors' collective expertise in physical literacy and inclusion. Particular attention is given to issues encountered when working with children with disabilities, while remaining relevant to broader populations. Key challenges were identified through engagement with the literature, iterative discussions among the authors, and brainstorming activities, allowing for the development of a shared understanding of the main barriers to inclusive physical literacy.

A preliminary review of the literature was conducted to identify the main challenges commonly discussed or empirically documented in both scholarly research and communities of practice. Documents and publications addressing “physical literacy” and “inclusion” were searched using Google Scholar, and additional relevant sources were identified through a snowballing process.

Building on this initial inventory of challenges related to the inclusivity of physical literacy, a structured brainstorming activity was conducted with the five authors of this paper. The process included one formal 2.5-hour synchronous session, followed by two rounds of asynchronous feedback through written comments. During the synchronous session, each participant first engaged in an individual idea-generation exercise guided by the following prompt: What challenges do you perceive, or have you previously encountered, regarding the inclusivity of physical literacy? Each challenge was documented on an interactive whiteboard. Challenges identified through the preliminary literature review were also added to the whiteboard.

The challenges were then presented to the group and briefly explained by the participant who had proposed them. This was followed by a collective discussion aimed at clustering and establishing connections among related challenges. Through this inductive process of collaborative sensemaking and thematic consolidation, four major categories of challenges were derived.

In a second phase, the team sought to identify levers for action regarding each category of challenges. Each participant independently formulated possible strategies or solutions addressing the previously identified challenges, guided by the question: What could help address these challenges and enhance the inclusivity of physical literacy? As in the previous phase, each idea was documented on the interactive whiteboard and discussed collectively to support collaborative sensemaking.

Following the synchronous session, the initial categorization was further refined by MB and submitted to the co-authors for two rounds of asynchronous comments. These rounds allowed the team to adjust the wording and definition of the categories.

Building on this combined knowledge base, the authors propose a set of interrelated challenges and levers for action aiming to contribute to ongoing discussions on how physical literacy can be more inclusively conceptualized and implemented across contexts.

## 3 Challenges and levers for action

Four major categories of challenges emerged from this process: 1) Redefining and Validating the Concept, 2) Mobilization within Educational Settings, 3) Bridging Knowledge Mobilization Gaps, and 4) Acknowledging Cultural and Systemic Impacts.

### 3.1 Redefining and validating the concept

A fundamental challenge to implementing inclusive physical literacy lies in its current definition, interpretation, and operationalization. The variety of definitions can pose a challenge, and it may discourage knowledge users from engaging with the concept, particularly non-native English-speaking professionals, since most of the definition work has taken place in English-speaking contexts (24). A scoping review by Martins et al. (2021) and a conceptual analysis by Young et al. (2020) both pointed out the diversity of perspectives concerning physical literacy, whether related to philosophical underpinnings, or core elements (6, 24). Some frameworks interpret physical literacy in ways that introduce a hierarchy among its dimensions, overemphasizing short-term motor-development objectives (6, 25), potentially marginalizing those whose abilities do not align with social norms (9, 22). Hebinck et al. (2023) highlight how professionals question terms such as “fundamental movement skills,” since tasks like jumping or running are far from universal (26). Moreover, the relationship between physical activity, physical literacy, and its dimensions remains largely theoretical (15), further complicating the operationalization of the concept.

To better realize its inclusive potential, the definitions, terms, and frameworks associated with physical literacy may benefit from continued critical examination, an objective already central to some studies (6, 9, 16, 24). Knowledge users are encouraged to clearly articulate what the concept represents for them and its intended impact (15): does it primarily target long-term health promotion or broader, holistic human flourishing? Acknowledging diverse forms of physical ability appears essential and could be operationalized through a greater emphasis on motivation and enjoyment (22). Further validation through rigorous longitudinal studies would help clarify long-term effects and relationships between dimensions (27, 28): does a physical-literacy-based approach support sustained engagement among young women during puberty? How does physical literacy contribute to culturally safe sport and leisure environments?

### 3.2 Mobilization within educational settings

Since young people in Canada are mostly introduced to physical activity at school, examining existing educational norms is essential to enhancing the inclusivity of physical literacy. The concept is often implied in targeted competencies but not explicitly named (29, 30). For example, Québec's physical education curriculum emphasizes competences such as adopting a healthy, active lifestyle, developing movement competence, and interacting with others (31), reflecting core dimensions of physical literacy without referencing it. This program dates back to 2001, around the time physical literacy was being reintroduced and redefined, which may partly explain its absence as an explicit framework. Nonetheless, this absence may contribute to challenges when attempting to implement a physical literacy-based approach within the curriculum. Although the current program proposes assessments with criteria, research suggests that, in practice, standardized tests based on normative values remain widely used (22, 32). More broadly, both the learning objectives and assessment practices tend to prioritize standardized and performance-oriented outcomes, overlooking individual variations and underemphasizing motivational dimensions central to physical literacy (22). Grauduszus et al. (2023) found that of 23 physical literacy assessments for youth, only 12 addressed all dimensions of the concept. When all dimensions are assessed, they are often scored and interpreted separately, fragmenting what is intended to be a holistic concept (33). More broadly, approaches to physical literacy may benefit from being reimagined to better account for contextualized learning experiences and to foster enjoyment and engagement, even before focusing on assessment and outcomes (34). If the goal is to cultivate lifelong engagement, informing a child at a young age that they have a “bad” physical literacy profile, or perform below peers, may be counterproductive, potentially undermining motivation and confidence.

Addressing this barrier may require a shift in practice. Competency development and assessments grounded in participant-centered observations represent a promising avenue (9, 34). Considering that physical literacy also develops through social interactions and shared experiences, a dimension more explicitly recognized in the Australian Physical Literacy Framework than in Canadian definitions, adopting mixed-ability groupings and accessible physical education approaches could help foster mutual aid and cooperation (9). Emphasizing holistic and ecosystemic approaches incorporating diverse contexts (school, community, home, health establishments) may also contribute to more sustainable outcomes (35). Strengthening collaboration between school communities, families, coaches, and professionals could facilitate implementation across settings, including schools, particularly for youth at greater risk of exclusion (26). Promoting educational approaches based on positive reinforcement and strengths-based perspectives may help create environments that prioritize enjoyment and personal development alongside performance (36). Recognizing and valuing effort and progress could further support engagement.

### 3.3 Bridging knowledge mobilization gaps

Significant gaps and biases remain in the scientific knowledge surrounding physical literacy. Most of the literature continues to reflect ableist tendencies, focusing on deficits rather than strengths (22), and there is limited actionable knowledge on implementation across settings, such as schools, sport clubs, or community centers (25). Opportunities for collaboration between scientific and professional communities remain limited (35), and the perspectives of marginalized groups or end-users are not always sufficiently represented, even when they are willing and able to contribute (22). Conversely, practice communities may have limited access to research-based knowledge and tools (35), resulting in knowledge that does not always circulate in ways that support meaningful and inclusive impact.

To mitigate these barriers, rethinking dissemination strategies through accessible resources (e.g., toolkits, online courses) and communities of practice appears valuable (2, 17, 22, 35). Collaborative knowledge production involving marginalized groups and practitioners may improve both relevance and application. Yi et al. (2019) highlight how community practitioners, including interns and part-time student workers, can meaningfully contribute to knowledge exchange (35). Enhancing training opportunities for all knowledge users (accessible, evidence-based, practical) also appears particularly important in inclusive physical activity and physical literacy (9, 10, 16, 22, 25, 26). Expanding training to include equity-related competencies could further support inclusive practices (37, 38), an approach that echoes the well-documented Pygmalion and Golem effects, whereby educators' expectations can shape perceptions and influence learners' performance and engagement (39). In other words, anticipated abilities can contribute to reinforcing existing inequities and limiting the development of physical literacy, especially for individuals in more vulnerable situations.

### 3.4 Acknowledging cultural and systemic impacts

Dominant norms and power structures, whether related to gender, culture or religion, significantly shape attitudes, opportunities, and practices in physical activity, often limiting its accessibility across contexts (37, 40). For girls and women, social expectations and stereotypes may reduce access to meaningful physical activity experiences (37, 38). In some socioeconomic contexts or situations, physical activity may be unaffordable, culturally inappropriate, or inconvenient in the face of other priorities (37, 41). Everyday elements such as dress codes, locker room configurations, scheduling, and the normalization of homophobic language can also represent barriers to participation (37, 41). The emphasis on individual responsibility within some definitions of physical literacy may further obscure the impact of collective values and systemic influences, as illustrated by Stevens et al. (2021) through a critical analysis based on Māori perspectives and Indigenous ways of knowing (42). Beyond norms, access to infrastructure remains uneven, particularly in rural areas, which may limit the capacity of even well-informed practitioners to implement inclusive practices (35).

To further support inclusive implementation, physical literacy initiatives may benefit from being more contextually and culturally situated. Reimagining physical spaces and questioning exclusionary norms related to performance, body image, gender expectations, and cultural biases represent important levers (37, 43). Expanding access for groups historically excluded from physical activity may contribute to more equitable participation and social justice (37, 40), and policymakers could play a key role by supporting inclusive local initiatives and promoting flexible, and diverse opportunities across contexts (37, 43). By way of illustration, such actions may include reconsidering the scheduling of activities to better account for religious holidays and diverse family obligations; or redesigning changing rooms to ensure the safety of all individuals, for instance through the provision of gender-neutral facilities. At a broader institutional level, these actions may also involve providing recurring funding envelopes for specific programs (e.g., adapted sport) or making inclusive practices a requirement for accessing public funding for programs, facilities, or municipalities. Regardless of the conceptual model adopted, addressing systemic inequities remains central: physical literacy, despite its inclusive potential, may fall short if these systemic and structural dimensions are not explicitly considered.

## 4 Discussion

The pursuit of inclusion within the framework of physical literacy entails navigating a series of complex, interrelated challenges that extend far beyond the individual level. Each challenge identified in this paper emerges from a cascade of determinants situated across multiple levels of society, from institutional policies and social norms to knowledge creation and practical implementation. This complexity underscores that fostering inclusivity in physical literacy cannot be achieved through isolated individual interventions or linear, prescriptive solutions.

To effectively promote inclusion, drawing on both the literature overview and the brainstorming activity, we propose three cross-cutting strategic orientations to guide future actions. First, it seems important to systematically identify and understand the range of factors influencing inclusive engagement in physical activity, such as socioeconomic status, power structures, social norms, accessibility of environments, dominant performance-oriented cultures, and the availability of adequate pedagogical resources. This comprehensive understanding will allow for the identification of leverage points where transformative action can occur, in favor of an inclusive, holistic interdisciplinary physical literacy approach. That identification alone is a massive undertaking: for example, a systematic review recently emphasized that we still lack quality data pertaining to sex and gender differences in physical activity and sedentary behavior (44).

Second, the co-creation and implementation of inclusive solutions should continue, and must actively involve a diversity of knowledge users, including practitioners, educators, researchers, policymakers, and community members with lived experience of exclusion, as previously proposed by others (9, 37). Their experiential knowledge is essential to ensure that interventions are contextually relevant, equitable, and sustainable.

Third, addressing these challenges requires an ecosystemic approach, one that recognizes the interdependence between the individual, institutional, and sociocultural levels, and integrates action across sectors such as health, education, sport, and community development (22). To better align physical literacy frameworks with this ecosystemic perspective, a major step might be to add a social dimension to the already well-established physical, cognitive, and affective dimensions, as some organizations (e.g., Australian Physical Literacy Framework) and authors have already done (45, 46). This will allow us to consider not only the person's relationships to others, but also the social influences and systemic barriers to physical literacy development. This perspective also invites reflection on how competencies are conceptualized within the physical literacy frameworks. While the development of fundamental movement skills is often emphasized, other forms of competencies, such as cognitive and social skills (e.g., problem-solving, decision-making, or interpersonal abilities), also play a role in supporting engagement and participation (22). Exploring how these dimensions could be more explicitly integrated may contribute to a more comprehensive and inclusive understanding of physical literacy.

A call to action is therefore warranted: to strengthen partnerships between academic researchers, practitioners, communities, and families; to support longitudinal and participatory research that examines inclusive practices in real contexts; and to embed social inclusion as a central, not peripheral, outcome in policy and program evaluation. By doing so, the field can move toward a more equitable, inclusive, and meaningful realization of physical literacy as a right and as a shared societal responsibility (see Figure 2B).

**Figure 2:**
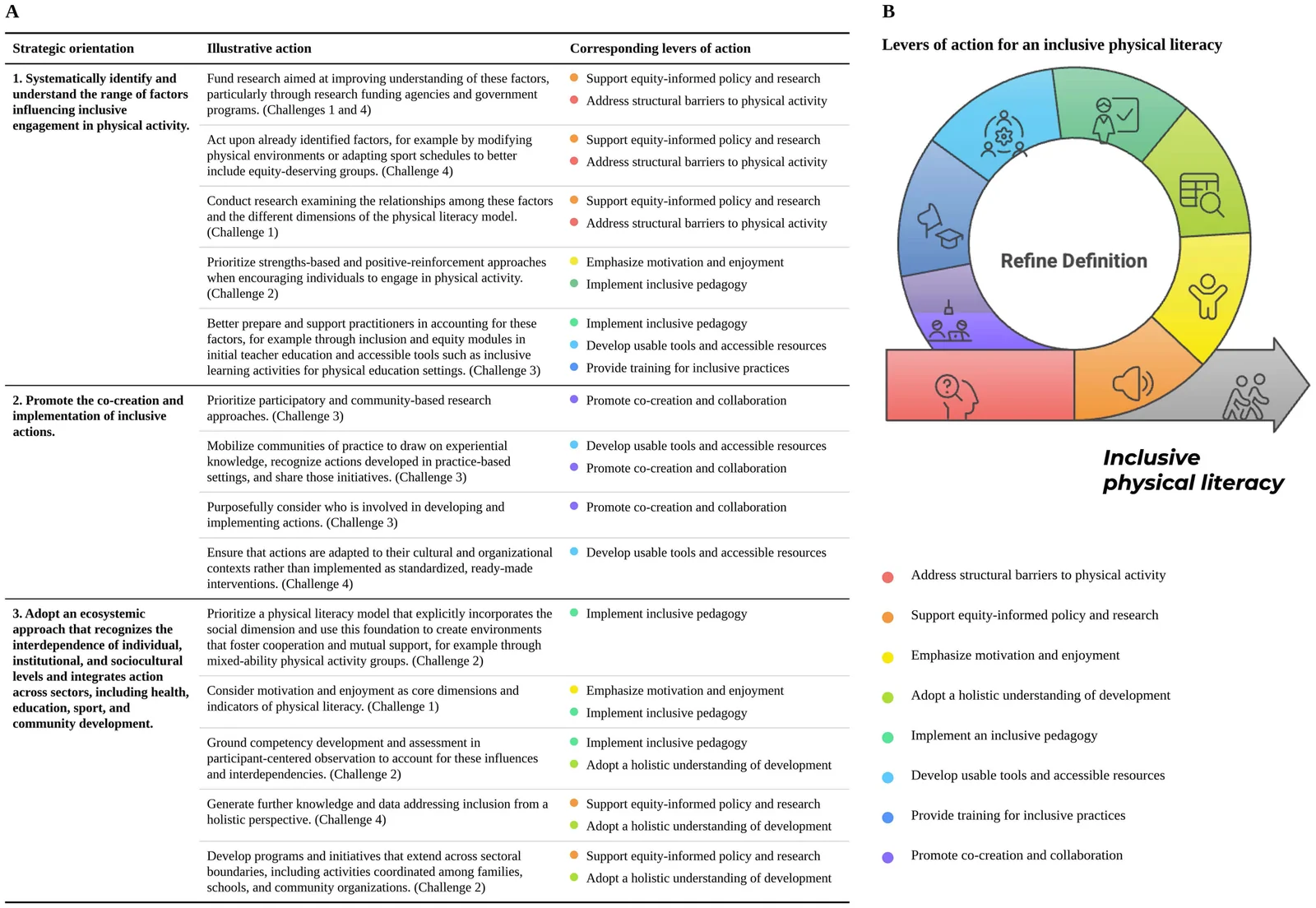
Levers of action for an inclusive physical literacy. Strategic orientations and illustrative actions **(A)** are mapped onto the eight levers of action they mobilize **(B)**. **(A)** Main strategic orientations identified in the study, along with illustrative actions and the corresponding levers of action. Numbers in parentheses refer to the challenges discussed in the text. Colored dots in the third column identify each lever and correspond to the segments and legend of panel B. These actions should be understood as components of systemic initiatives. Although individuals (e.g., practitioners, educators, researchers, policymakers, and community members with lived experience of exclusion) can contribute to their implementation, organizations and governments also have a central role in developing action plans, setting priorities, adopting institutional and public policies to support sustained and coordinated action. **(B)** Visual representation of key actions supporting inclusive physical literacy. The central element highlights the need to refine the definition of physical literacy, while the circular structure reflects the multilevel actions proposed through the paper: *address structural barriers to physical activity, support equity-informed policy and research, emphasize motivation and enjoyment, adopt a holistic understanding of development, implement an inclusive pedagogy, develop usable tools and accessible resources, provide training for inclusive practices, and promote co-creation and collaboration.* The arrow indicates the intended outcome of inclusive physical literacy, understood as supporting lifelong well-being and human flourishing for all.

## 5 Conclusion

This work moves beyond documenting familiar barriers by organizing the challenges facing physical literacy into four cross-cutting categories and proposing systemic, multilevel levers that address the entire ecosystem, extending prior work on physical literacy and inclusion toward a broadly inclusive and holistic approach rather than a population-specific one. As physical literacy continues to expand its reach, addressing these challenges is crucial. This is a call to action for all knowledge users (e.g., educators, policymakers, community leaders, families, and youth) to collaborate intentionally and proactively to address these critical issues. Seizing this growing momentum around physical literacy presents a unique opportunity to promote equity, representation, and inclusion within our communities and beyond. This also invites a shift in how physical literacy is conceptualized, moving beyond an individual focus to more fully acknowledge the social, cultural, and systemic conditions that shape engagement in physical activity. By acting collectively, we can ensure that all Canadians have better opportunities to flourish and benefit fully from the transformative potential of physical literacy.

## Data Availability

The original contributions presented in the study are included in the article/Supplementary Material, further inquiries can be directed to the corresponding author/s.
